# Exocytotic fusion pore under stress

**DOI:** 10.15698/cst2020.09.230

**Published:** 2020-08-10

**Authors:** Helena Haque Chowdhury, Robert Zorec

**Affiliations:** 1 Laboratory of Cell Engineering, Celica Biomedical, 1000 Ljubljana, Slovenia.; 2 Laboratory of Neuroendocrinology – Molecular Cell Physiology, Institute of Pathophysiology, University of Ljubljana, Medical Faculty, 1000 Ljubljana, Slovenia.

**Keywords:** exocytosis, fusion pore, SNARE proteins, secretory vesicle, fusion pore stability

## Abstract

Exocytosis is a universal process of eukaryotic cells, consisting of fusion between the vesicle and the plasma membranes, leading to the formation of a fusion pore, a channel through which vesicle cargo exits into the extracellular space. In 1986, Rand and Parsegian proposed several stages to explain the nature of membrane fusion. Following stimulation, it starts with focused stress destabilization of membranes in contact, followed by the coalescence of two membrane surfaces. In the next fraction of a millisecond, restabilization of fused membranes is considered to occur to maintain the cell's integrity. This view predicted that once a fusion pore is formed, it must widen abruptly, irreversibly and fully, whereby the vesicle membrane completely integrates with and collapses into the plasma membrane (full fusion exocytosis). However, recent experimental evidence has revealed that once the fusion pore opens, it may also reversibly close (transient or kiss-and-run exocytosis). Here, we present a historical perspective on understanding the mechanisms that initiate the membrane merger and fusion pore formation. Next, post-fusion mechanisms that regulate fusion pore stability are considered, reflecting the state in which the forces of widening and constriction of fusion pores are balanced. Although the mechanisms generating these forces are unclear, they may involve lipids and proteins, including SNAREs, which play a role not only in the pre-fusion but also post-fusion stages of exocytosis. How molecules stabilize the fusion pore in the open state is key for a better understanding of fusion pore physiology in health and disease.

## INTRODUCTION

**Figure 1:**
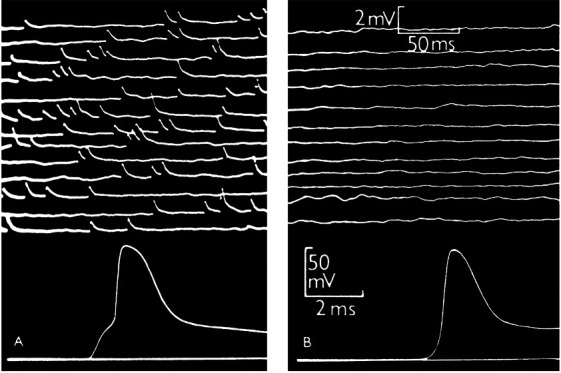
FIGURE 1: Intracellular recording from single muscle fibre of frog. **(A)** At the motor end-plate. The upper part shows spontaneous miniature end-plate potentials, which are localized at a junction and arise from sudden discharge by a motor nerve ending of packets of acetylcholine, each containing thousands of molecules. The lower part shows a single response to a nerve impulse, which was started by electric shock at the beginning of the trace; the first step of the response is large end-plate potential resulting from synchronous delivery of a few hundred packets of acetylcholine, this leading to full size action potential. **(B)** Traces recorded in the same muscle fibre, 2 mm away from the end-plate. The upper part shows much attenuated and barely recognizable residues of miniature end-plate potentials. The lower part shows propagated action potential, delayed by conduction over 2 mm distance and not preceded by the end-plate step. From [1]

**Figure 2:**
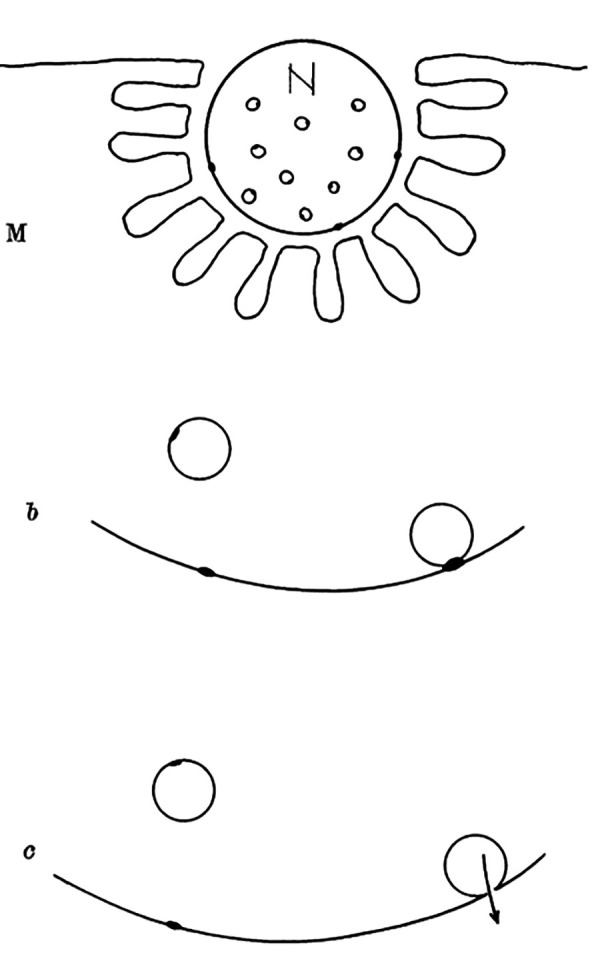
FIGURE 2: Diagram explaining quantal secretion of neurotransmitters by exocytosis of synaptic vesicles. Reaction molecules (fusion proteins) are indicated by black dots on vesicular and nerve membranes; a nerve impulse greatly increases the number of reactive sites in the terminal membrane by allowing calcium ions to penetrate it. N, motor nerve terminal; M, end-plate region of a muscle fibre. This scheme was presented by J. del Castillo and B. Katz at a Symposium at Gif-sur-Yvette in July 1955 [73].

Although it is more than seven decades since Sir Bernard Katz and colleagues started to investigate synaptic vesicle fusion with the plasma membrane at the end-plate (the neuromuscular junction), progress in understanding this ubiquitous process of eukaryotic cells has been slow. The original observation of “biological noise” (**Fig. 1**) at the stimulated neuromuscular junction, represented by miniature end-plate potentials [1], already recorded in 1948 at the skeletal muscle membrane [2], was instrumental for the formulation of the “quantal theory of neurotransmitter release”. This was recognized widely by awarding the Nobel Prize in 1970 to Sir Bernard Katz, Ulf von Euler and Julius Axelrod, “for their discoveries concerning the humoral transmitters in the nerve terminals and the mechanism for their storage, release and inactivation” [3]. In his Nobel Laureate lecture, Sir Bernard Katz outlined the events that regulate neurotransmitter release: depolarization opens specific “calcium gates” in the terminal axon membrane, leading to an influx of calcium ions to initiate the “quantal release reaction”. Based on biochemical and structural data available at that time, he also considered the hypothesis “that the quanta of transmitter molecules are enclosed within synaptic vesicles which frequently collide with the axon membrane, and that calcium causes attachment and local fusion between vesicular and axon membranes” [4]. He postulated a structure at the plasmalemma where vesicles frequently collide, depicted as black dots in **Fig. 2**.

A further step towards understanding the nature of events that occur during the merger of the vesicle membrane with the plasmalemma was the consideration that this process consists of distinct steps [5]. (i) Stable membrane apposition: Rand and Parsegian argued that most membranes do not fuse, however, when membrane fusion occurs, there are exceptional, strictly controlled conditions to allow specific membrane pairs to merge. (ii) Triggering of membrane fusion was considered to be associated with an increase in intracellular calcium. (iii) Contact fusion was considered to involve a closer functional merger of membranes, closer than the stable apposition achieved by most membranes. (iv) Focused destabilization was considered in such a way that the structure of the contacting membranes must destabilize, and that destabilization must be restricted to the contact area to ensure that the fusion of membranes is “leakless,” i.e. that the correct aqueous compartments mix. (v) Membrane coalescence: the destabilized structure was considered to lead to the coalescence of two membrane surfaces. (vi) Restabilization: the fused membrane was thought to restabilize quickly to maintain the integrity of the membrane and the cell. Importantly, Rand and Parsegian noted that stages (iv), (v), and (vi), and perhaps (iii), appear extremely rapidly (in microseconds), even if the events leading to them are slow. This latter view prompted the idea that once the fusion pore is formed, it must widen abruptly and fully. This mode of exocytosis is often termed full fusion (irreversible) exocytosis, meaning that the vesicle membrane completely integrates with and collapses into the plasma membrane and is retrieved only by regulated endocytosis.

**Figure 3:**
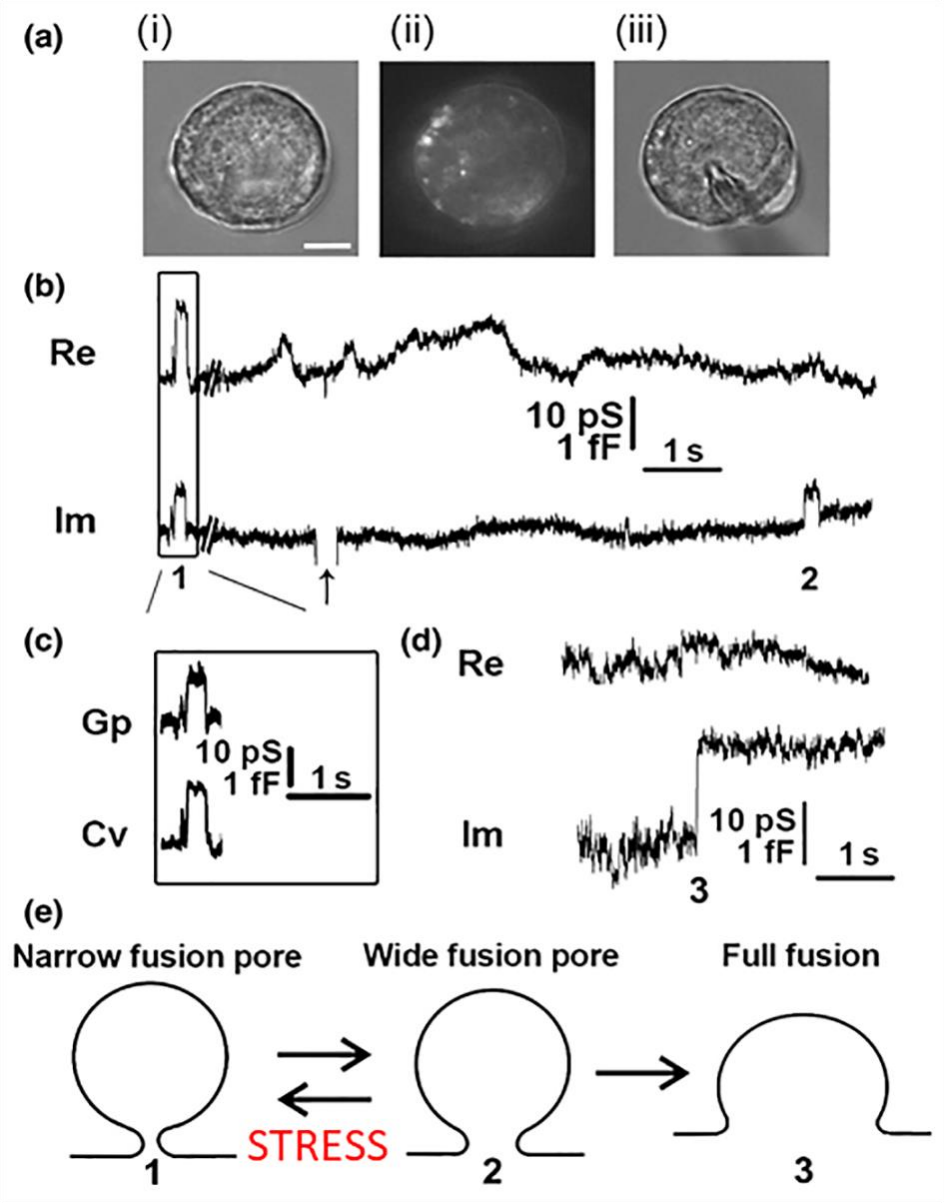
FIGURE 3: Cell-attached membrane capacitance (C_m_) measurements on pituitary lactotrophs show transient (reversible) and full fusion exocytotic events. **(A)** Lactotrophs co-transfected with hyperpolarization-activated and cyclic nucleotide-gated 2 pDNA and EGFP pDNA were used for the C_m_ measurements in the cell-attached configuration [12]. **(Ai–Aiii)** All measurements were performed only on lactotrophs exhibiting EGFP fluorescence, as shown for the same cell under transmitted light **(Ai)**, under epifluorescence **(Aii)**, and during the measurement **(Aiii)**. Scale bar, 3 μm. **(B)** An epoch from a representative recording showing the reversible steps in the Im part of the admittance trace. Reversible events in Im, which likely represent transient exocytotic events (transient fusion pore opening), either exhibited projections to the Re part of the admittance trace (1) or not (2). The arrow points to the calibration pulse in Im, which was triggered manually to ensure the correct phase angle settings. **(C)** Calculated vesicle capacitance (Cv) and fusion pore conductance (Gp), a measure of fusion pore geometry, for all reversible events with projections [74]. **(D)** A representative example of irreversible upward steps in the Im trace and the corresponding Re trace (3), which likely denotes a full fusion exocytotic event. **(E)** A scheme of the stages that a vesicle must pass to completely fuse with the plasma membrane: from narrow fusion pore formation (1), to widening of the fusion pore (2), and finally to full fusion of the vesicle membrane and the plasma membrane (3). From Ref. [74].

In contrast to this mode of exocytosis, recent data revealed that the fusion pore may enter a stable, dynamically regulated state, with a diameter ranging between subnanometres to several hundred nanometres [6, 7]. These fusion pores can then undergo either reversible constriction/closure or can widen again, consistent with the original observation that synaptic vesicles fuse with, and re-form from, the membrane of the nerve terminal during stimulation and that the re-formed vesicles can store and release transmitter [8]. This mode of exocytosis was named as kiss-and-run exocytosis [9]. This terminology has been considered in part by biophysical patch-clamp experiments to monitor membrane currents and membrane capacitance, a parameter linearly related to changes in the membrane area. This technique permits direct determination of the fusion pore opening and closing [10] and has been used in many secretory cells, including chromaffin, mast, pancreatic, plant and pituitary cells [10–17], where it has revealed that in addition to full fusion, the fusion pore, once open, can reversibly close or constrict (**Fig. 3**). These observations established that the fusion pore opening is a real biological phenomenon where the fusion pore conductance, a measure of how narrow is the fusion pore, can be determined under different physiological conditions. By studying giant secretory vesicles, currents through the open fusion pore were measured [18]. Simultaneous electrical and optical measurements revealed that the formation of the fusion pore precedes vesicle swelling [19]. Reversible closure (transient opening) of the fusion pore may occur several times, and it was observed in different cell types including chromaffin and pituitary cells, plant protoplasts and astrocytes [6, 13, 17, 20–22]. Hence, it is highly unlikely that the same vesicle “runs” away from the plasmalemma each time the fusion pore closes. Therefore, this form of exocytosis was termed reversible or transient fusion pore opening or “reversible or transient exocytosis” [6, 17]. The fusion pore opening can result in a very narrow pore diameter, smaller than the actual molecular size of the vesicle cargo, thus this cargo cannot be secreted to the cell exterior. In this case, the mode of exocytosis was termed “unproductive exocytosis” [23]. **Fig. 3E** indicates transitions between the intermediate fusion stages of an exocytotic vesicle. The initial narrow fusion pore can reversibly widen and return to a narrow or even a closed state, however at some point in time it may also transit to a fully wide state (full fusion).

Transitions between the stages that the vesicle passes during membrane merger appear to depend on vesicle size [7, 24, 25], and it is generally acknowledged that these transitions are regulated by proteins [7, 26], which may also form the fusion pore walls [27–29]. The fusion pore was also modelled to be exclusively lined by lipids [30]; however, it is more likely to be a mixture of proteolipids [31, 32]. Although it is experimentally difficult to record single-vesicle fusion in synapses, which are specialized for fast signalling, and the vesicles are relatively small, it appears that fusion pores of these vesicles predominantly exhibit fully open fusion pores, with a small fraction of events exhibiting transient fusion pore openings [33–35]. In these vesicles, the opening of the fusion pore follows the calcium trigger in a millisecond and is considered to be associated with the SNARE (soluble NSF-attachment protein receptors) protein complex, which is thought to initiate exocytotic membrane fusion [36]. In the next sections, we highlight the nature of pre- and post-fusion mechanisms of the vesicle membrane merger with the plasmalemma.

## PRE-FUSION MECHANISMS

The initial stages of membrane fusion were considered to consist of focused destabilization, whereby a spatially restricted structure of the contacting membranes must destabilize to allow membrane coalescence [5]. Based on these predictions, where the proximity between membranes should be much tighter than the ordinary membrane-to-membrane distance, typically determined in part by the repulsion of negatively charged membrane surfaces [5], a suitable mechanism may involve proteins that span two membranes and interact to bring the two membranes closer. Among many recognized candidates, proteins termed SNAREs have received attention. Their discovery was part of the 2013 Nobel Prize in Physiology or Medicine, awarded to James E. Rothman, Randy W. Schekman and Thomas C. Südhof “for their discoveries of machinery regulating vesicle traffic, a major transport system in our cells” [37]. Whereas the coworkers of T.C. Südhof discovered signals that coordinate the timing of when the synaptic vesicles release their cargo in neurons, the coworkers in the lab of R. Schekman described a set of genes required for vesicle transport. The research from the group of J.E. Rothman studied the SNAREs, a group of three proteins: vesicle-associated membrane protein (VAMP)/Synaptobrevin, synaptosome-associated protein of 25 kDa or 23 kDa (SNAP-25 or SNAP-23) and syntaxin. These three interacting SNARE proteins, localized to the presynaptic region, had previously been identified and studied by researchers (including Kimio Akagawa, Richard Scheller, Reinhard Jahn and Pietro de Camilli), but their function was largely unclear. These proteins were found to be ideally located across the vesicle and the plasmalemma; VAMP/Synaptobrevin is a vesicle protein, whereas SNAP-25 and syntaxin were found at the plasma membrane. This enabled the generation of the SNARE hypothesis proposing that target and vesicle SNAREs (t-SNAREs and v-SNAREs) are engaged in vesicle fusion through several sequential steps, including hemifusion, an early stage of membrane coalescence according to Rand and Parsegian. This stage was visualized in larger, experimentally more accessible vesicles [38] and is likely playing a role in vesicle docking (anchoring to the specific sites, predicted already by Sir Bernard Katz; **Fig. 2**). These initial steps involve the activation of a local mechanical stress to promote membrane fusion, leading to the merger of the vesicle and the plasma membranes. This interpretation, especially the local mechanical stress during focal apposition of fusing membranes, was influenced by bulk biochemical studies, which revealed that the ternary SNARE complex (an association of the three types of SNARE molecules) is a thermally very stable structure [39]. This led to the concept that once the ternary SNARE complex is formed, it may not be easily disassembled unless special enzymes with provision of energy in the form of ATP are in place [40]. Hence, this stable SNARE complex can be considered a molecular nucleus generating a focal mechanical stress, predicted by Rand and Parsegian in 1986. In the next stages, this focal structure was predicted to enable vesicle cargo discharge in an all-or-none-fashion, as envisioned by the “quantal release of neurotransmitter” considered by Sir Bernard Katz.

Anchoring of the SNARE proteins to the specific sites at the plasma membrane is related to membrane rafts [41], structures with enriched amounts of cholesterol, the most common steroid in humans and a major constituent of the cell membrane, which affects membrane fusion [42]. Although many experiments addressed this topic in the past [43–47], direct demonstration of how cholesterol affects distinct stages of regulated exocytosis, especially the dynamics of single fusion pore gating is still unclear. It is known that an increase in membrane cholesterol increases the probability of a vesicle fusing with the plasma membrane [48]. These findings are consistent with the view that negative curvature of cholesterol promotes the first steps of the membrane merger pathway [45, 49, 50], the formation of restricted sites in the membrane, where focused destabilization, according to Rand and Parsegian, can be initiated. However, the question was raised as to whether the increased curvature due to enhanced cholesterol content in membrane rafts is sufficient to represent a native mechanism of membrane fusion, without any additional presence of proteins such as SNAREs [50].

The presence of membrane rafts, and especially the anisotropic negative curvature shape of cholesterol, has been viewed as functionally affecting the ability to shape the membrane curvature locally, which may influence the fusion pore formation and its stabilization [24, 45]. However, a less explored mechanism of the role of membrane rafts in membrane fusion mechanisms is that represented by the generation of tension between intermembrane domains with heterogeneous structure [51, 52], which may play a role in post-fusion mechanisms.

## POST-FUSION MECHANISMS

Recently accumulated evidence that the fusion pore may enter into a relatively stable but dynamically regulated state [6, 7] indicates that there are forces that control fusion pore opening and closure. When these forces are balanced, the fusion pore exhibits a stable fusion pore diameter. Although the mechanisms of fusion pore stabilization are unknown, vesicle size itself and several proteins, including actin, syntaxin, synaptotagmin, Munc 18-1, dynamin, CAPS and others appear to play a role [7, 24–26, 29, 53–56].

The cortical actin cytoskeleton was considered a barrier for exocytotic vesicle delivery to the plasma membrane [57–59]. Subsequently, however, actin was found to be required for regulated exocytosis [60], in particular for widening of fusion pores with diameters exceeding 100 nm [7], an intermediate structure through which vesicle membrane transfer to the plasmalemma is manifested as vesicle shrinkage [61–63], influenced by the osmotic pressure difference between the intracellular and the extracellular solution and actin-providing membrane tension to reel in the membrane of the fusing vesicles [62, 63]. The transition of the membrane fusion pore with a completely widened diameter arguably results from competition between calcium-dependent activation of exocytotic machinery and dynamin-dependent fusion pore closure [38, 55].

**Figure 4:**
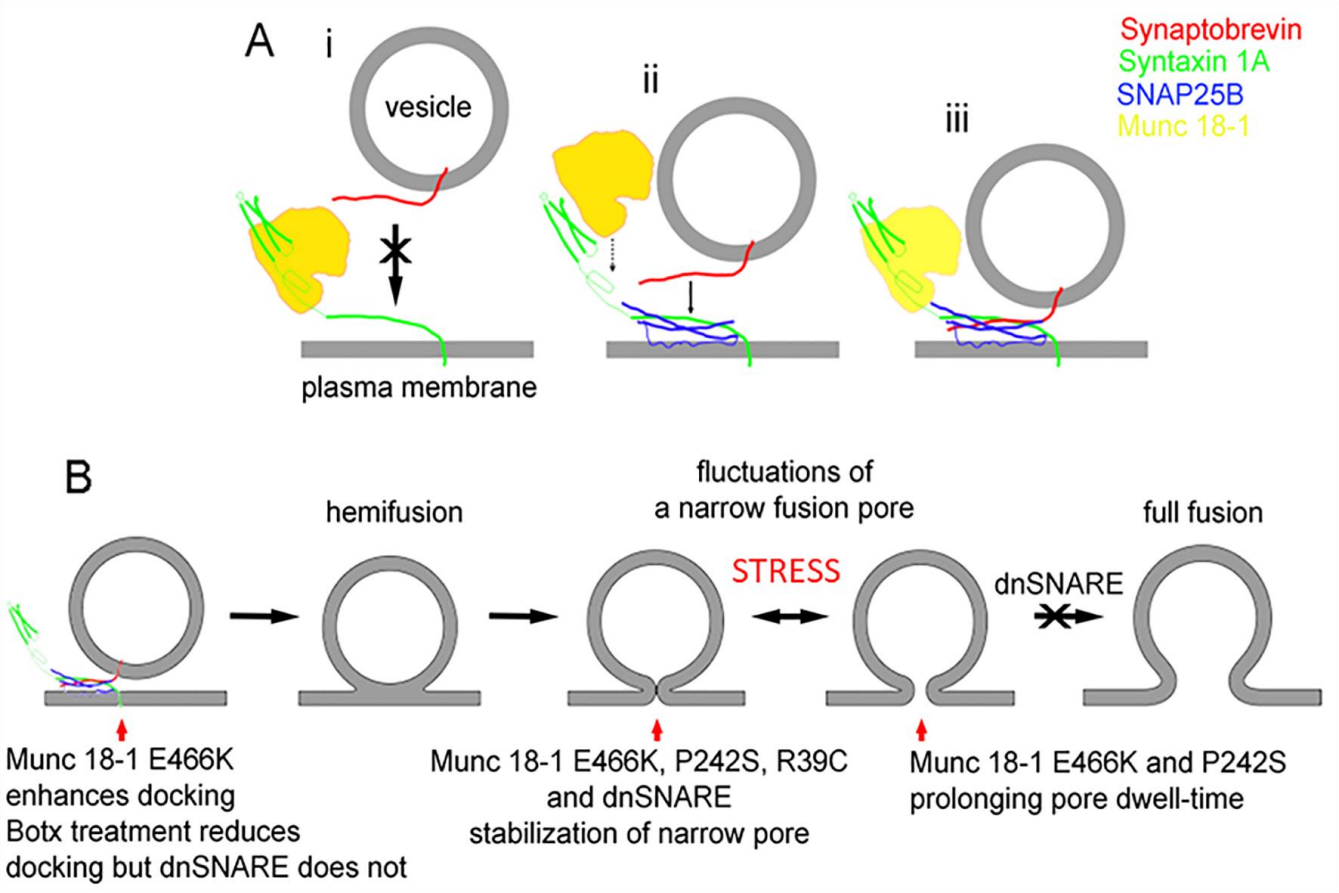
FIGURE 4: A diagram displaying the interaction of Munc18-1 an SNARE proteins along the stages of exocytosis. **(A)** The interaction between Munc18-1 and SNARE proteins, indicating that Munc18-1 favours the formation of the ternary SNARE complex where synaptobrevin binds preferably with the binary complex between syntaxin and SNAP25. **(B)** The stages from docking/priming, hemifusion state, leading to fusion pore formation and full fusion, with the action of respective Munc18-1 mutants and dnSNARE peptides. From Ref [26].

Interestingly, SNARE proteins not only play a role in pre-fusion but also during post-fusion mechanisms. Indeed, when the function of the accessory SNARE protein Munc 18-1 was studied at the level of a single-vesicle interaction with the plasmalemma, using the high-resolution patch-clamp capacitance technique, it was revealed that there are multiple sites where the SNARE complex controls the exocytotic intermediates [26]. In the study using endocrine pituitary cells, Munc 18-1 mutants were transfected into secretory cells to affect the interaction of Munc 18-1 with syntaxin1 (Synt1, R39C), with Rab3A (E466K), and Mint proteins (P242S). In comparison with wild-type Munc 18-1, the mutant Munc 18-1E466K increased the frequency of the unitary fusion events, consistent with the view that Rab3A protein, a small GTPase [64], facilitates vesicle docking at the plasma membrane. Whereas the other Munc 18-1 mutants (R39C and P242S) increased the fusion pore dwell-time, all the mutants stabilized the geometry of a narrow diameter fusion pore, indicating that transition of a narrow diameter fusion pore into a more widely or completely open one is hindered by all these mutants. Single-molecule atomic force microscopy experiments revealed that wild-type Munc 18-1, but not Munc 18-1R39C, abrogates the interaction between synaptobrevin2 (Syb2, a v-SNARE protein) and Synt1 binary *trans*-complexes [26]. Importantly, neither form of Munc 18-1 affected the interaction of Syb2 with the preformed binary *cis*-Synt1-SNAP25 complexes at the plasmalemma, revealing that Munc 18-1 performs a proofing function by inhibiting tethering of Syb2-containing vesicles solely to Synt1 at the plasmalemma and promoting vesicular tethering via Syb2 to the preformed binary *cis*-complex of Synt1-SNAP25 (**Fig. 4A**).

The studies using atomic force spectroscopy of single SNARE molecule interactions revealed the disassembly properties of the ternary SNARE complex to be in the time domain of 0.2 to 2 s [65], which is consistent with fusion pore kinetics [22, 26]. Therefore, assembly and disassembly of the ternary SNARE complex that is influenced by SNARE accessory proteins, can take place not only at vesicle docking, which facilitates the vesicle membrane merger with the plasma membrane, but also at other later intermediate stages of exocytosis (**Fig. 4B**), such as the transient widening of the fusion pore and regulation of the fusion pore dwell-time, both processes leading to full fusion, a complete integration of vesicle membrane into the plasma membrane [66].

**Fig. 4B** depicts the stages that a vesicle undergoes to reach the capacity to fully integrate with the plasma membrane (full fusion). The last step in the sequence of transitions is indicated to be inhibited by the dominant-negative domain of synaptobrevin 2 protein peptide (dnSNARE). This peptide has been considered to block the formation of the ternary SNARE complex and was hence used to block vesicle-based exocytosis, especially in astrocytes, a type of neuroglial cells [67]. Although it is still debated whether gliotransmission, a process that depends on the rapid detection of synaptic activity by astrocytes, is present *in vivo*, ample evidence indicates that regulated exocytosis is present in astrocytes [22, 68] but with much slower kinetics [69] than in neurons. The paradigm of rapid transmitter release from glial cells to be present *in vivo* critically depends on experiments in transgenic mice expressing the dnSNARE peptide. However, the mechanism of action of this peptide at the level of a single vesicle was recently described [22], revealing that the action of this peptide is not at the pre-fusion stage, the stage of the formation of the SNARE complex, but at the stage when the fusion pore is already open [22]. Moreover, when botulinum neurotoxins cleaving Syb2 and SNAP-23 were transfected into cells, the frequency of unitary exocytotic events was reduced as expected if the formation of ternary SNARE complex is needed for the vesicle fusion with the plasma membrane. However, when the dnSNARE peptide was introduced into cells, the frequency of exocytotic events was unaffected, consistent with the view that dnSNARE peptide acts at the post-fusion stage of regulated exocytosis, constricting the pore to a very narrow diameter. This dnSNARE-mediated fusion pore stabilization in astrocytes [22], which is considered release unproductive [23], was observed in lactotrophs transfected with Munc 18-1 mutants [26].

Mutant SNARE-interacting proteins (Munc18-1, Rab3A, and Mints), as well as a dominant-negative synaptobrevin peptide dnSNARE, stabilize fusion pores with subnanometre diameter [22, 26], indicating that the SNARE complex in the “unzippered” state (i.e. SNARE proteins are unable to interact to form a complex) is unable to oppose the forces constricting the fusion pore, clearly a post-fusion mechanism. The nature of these forces is generally not known, but dynamin is a strong candidate here [38, 55]. Moreover, lipids can also regulate the fusion pore [32], including phosphatidic acid produced by phospholipase D1. It has been shown that phosphatidic acid helps in recruiting and/or activating the exocytotic protein machinery. It also directly affects the membrane curvature and thus favours membrane rearrangements as required for membrane fusion [70]. A similar mechanism was previously proposed for phosphatidylinositol 4,5-bisphosphate (PIP2) [71] and for cholesterol [50]. In the latter case, as already discussed in the previous section, membrane rafts enriched with cholesterol may generate a force on the neighbouring membrane regions with heterogeneous membrane structure [52], and these forces may play a role in membrane fusion [42]. However, direct demonstration of such a role of cholesterol in membrane fusion has only recently been addressed [72].

## CONCLUSIONS

In summary, regulated exocytosis is a multistage process. In the past, the stages that a vesicle passes during exocytosis were considered to occur abruptly at specific sites to minimize the leak of cytosolic components during membrane destabilization, hence the process was considered to be irreversible, ending in full fusion. Recent data obtained by monitoring vesicle fusion at the single-vesicle level revealed that fusion pores exhibit remarkable stabilization: not only when they are smaller than a nanometre but also at several hundred nanometres of opening. These stable fusion pore diameters reflect a balance between forces that open and close the fusion pore. Although proteins (especially SNARE proteins) are considered the major players in the regulation of fusion pore diameter, there is evidence that lipids, including cholesterol, play a role in pre- and post-fusion mechanisms. Therefore, the membranes engaged in fusion pore formation exhibit mechanical stress during initiation of the process but also at the stage when the pore is already open.

## Abbreviatons:

dnSNARE: – dominant-negative domain of synaptobrevin 2 protein peptide;
SNARE: – soluble NSF-attachment protein receptors.
